# Anthocyanin Recovery from Grape by-Products by Combining Ohmic Heating with Food-Grade Solvents: Phenolic Composition, Antioxidant, and Antimicrobial Properties

**DOI:** 10.3390/molecules26133838

**Published:** 2021-06-24

**Authors:** Marta Coelho, Sara Silva, Eduardo Costa, Ricardo N. Pereira, António Sebastião Rodrigues, José António Teixeira, Manuela Pintado

**Affiliations:** 1 CBQF-Centro de Biotecnologia e Química Fina-Laboratório Associado, Escola Superior de Biotecnologia, Universidade Católica Portuguesa, Rua Arquiteto Lobão Vital 172, 4200-374 Porto, Portugal; mccoelho@ucp.pt (M.C.); snsilva@ucp.pt (S.S.); emcosta@ucp.pt (E.C.); 2 CEB-Centre of Biological Engineering, University of Minho, 4710-057 Braga, Portugal; rpereira@deb.uminho.pt (R.N.P.); jateixeira@deb.uminho.pt (J.A.T.); 3 Centre for Toxicogenomics and Human Health, Genetics, Oncology and Human Toxicology, NOVA Medical School/Faculdade de Ciências Médicas, Universidade Nova de Lisboa, 1169-056 Lisbon, Portugal; sebastiao.rodrigues@nms.unl.pt

**Keywords:** grape by-products, ohmic heating, conventional methods, biological properties, phenolic compounds, anthocyanins, antimicrobial, antioxidant activity

## Abstract

Usually, wine-making by-products are discarded, presenting a significant environmental impact. However, they can be used as a source of bioactive compounds. Moreover, consumers’ increasing demand for naturally nutritious and healthy products requires new formulations and food product improvement, together with sustainable, environmentally friendly extraction methods. Thus, this work aimed to compare ohmic heating (OH) with conventional methodology (CONV), using food-grade solvents, mainly water, compared to standard methanol extraction of anthocyanins. No significant differences were found between the CONV and OH for total phenolic compounds, which were 2.84 ± 0.037 and 3.28 ± 0.46 mg/g DW gallic acid equivalent, respectively. The same tendency was found for antioxidant capacity, where CONV and OH presented values of 2.02 ± 0.007 g/100 g and 2.34 ± 0.066 g/100 g ascorbic acid equivalent, respectively. The major anthocyanins identified were malvidin-3-*O*-acetylglucoside, delphinidin-3-*O*-glucoside, petunidine-3-*O*-glucoside, cyanidin-3-*O*-glucoside, and peonidine-3-*O*-glucoside. These extracts displayed antimicrobial potential against microorganisms such as *Yersinia enterocolitica*, *Pseudomonas aeruginosa*, *Salmonella enteritidis*, methicillin-sensitive *Staphylococcus aureus*, a methicillin-resistant *Staph. aureus* (MRSA), and *Bacillus cereus*. In conclusion, OH provides similar recovery yields with reduced treatment times, less energy consumption, and no need for organic solvents (green extraction routes). Thus, OH combined with water and citric acid allows a safe anthocyanin extraction from grape by-products, thus avoiding the use of toxic solvents such as methanol, and with high biological potential, including antimicrobial and antioxidant activity.

## 1. Introduction

The wine-making process produces a large number of by-products that have a significant environmental impact. This process generates a high amount of solid organic waste, namely stalks, pomace (including skins, seeds, grape pulp l) and lees, which may be disposed of or beneficial use [1,2]. Nevertheless, these by-products can also be used as a source of bioactive compounds, such as dietary fiber, grape seed oil, hydrocolloids, and phenolic compounds, which might be applied by the agri-food and feed industries promoting economic value. Its reuse follows the actual circular economy concept imposed by the European Union. According to it, strategies for smart, sustainable, and inclusive growth must be adopted, promoting environmental protection [3,4,5]. 

Directive 2008/98/EC of the European Parliament and of the Council of 19 November 2008 established a legal framework for treating waste in the European Union, emphasizing the importance of proper waste management, recovery, and recycling techniques to reduce the environmental and human health impact [6]. Value-added compounds could be isolated from the by-products to be used either as natural ingredients for the formulations of functional foods or as additives. These by-products have drawn the attention of scientists and the food industry. Traditionally, grape pomace has been used to produce wine beverages, nutrient colorants, and grape oil. More recently, research has concentrated on creating different value-added products, e.g., bioactive compounds, primarily phenols, healing of tartaric acid, and the production of flours [5,7]. 

These wine industry by-products are frequently undervalued but represent a possible source of bioactive compounds, such as polyphenols, that could be applied in many industries. Phenolic substances from grapes, including anthocyanins, are reduced at the skin and seeds, more precisely, the portion that remain as pomace after the processing of grapes [2,7,8]. 

Anthocyanins belong to the flavonoid class and represent the most significant set of water-soluble plant pigments [2,9]. Anthocyanin’s color depends on the solvent’s pH; the red-color anthocyanins are stable at lower pH (3.5–4) [7,9,10].

As they are localized in black grape peels, anthocyanins are usually extracted (30 to 40%) through wine-making operations. However, previous research has shown that the anthocyanin content of a given cultivar is not necessarily positively linked with anthocyanin concentration in the resulting wine. The lack of association was due to the partial preservation by cell-wall polymers of these anthocyanins in the skin cells [2,11,12].

Various methods have been developed to extract bioactive compounds, and their effects on the preparation and functionality of extracts from agro-industrial wastes have been evaluated. These commonly applied methods use harmful and toxic compounds, restricting the use of grape by-product extracts [2,7,13]. Nevertheless, given the need for sustainability, the political agenda is fostering the development of “clean label” processes toward the reduction of environmental impacts and a strong bioeconomy. In addition, the conversion of wine-making by-products into added-value products could be possible through the development of environment-friendly technologies. New technologies, such as microwave-, ultrasound-, and ohmic heating (OH)-assisted extraction, have been used to improve the recovery of bioactive compounds from food samples [2,8,14]. These methods have attracted significant attention from the scientific community. In particular, OH is a thermal process where an alternating electric flow is forced to pass through the food materials [2]. The thermal effect needed to assist the extraction process is rendered internally due to the passage of electric flow through the materials (Joule effect). The general purpose of OH technology is to improve food, cosmetics, and pharmaceutical products that are safe and beneficial to human health [2,15,16]. Despite these advantages, the OH application can be impaired by products’ physical and chemical properties, such as poor electrical conductivity due to higher fat or sugar contents in their composition [16]. 

El Darra and colleagues performed an assisted extraction by pulsed OH from red grape pomace. The authors explored the pulsed OH effects on cell membrane damage to increased polyphenol recovery. They also studied the effects of the electrical field strength, temperature, and the proportion of ethanol/water used. Pre-treatment caused cell membrane permeabilization. In addition, pulsed OH, which was used as a pre-treatment, increased the recovery kinetics of TPC. Other researchers reinforce high yields of recovery of phytochemicals obtained by OH from black rice bran. They suggest OH as a promising technology to extract anthocyanins with a future application in the production of natural colorants [17].

Pereira et al. [18] explore how ohmic heating (OH) influences phytochemical components recovery from colored potatoes (*Solanum tuberosum* L. var. *Vitelotte*) using moderate electric fields. Their results reveal that low-energy electrical fields and thermal effects may be integrated and adjusted into a single phase of treatment by recovering anthocyanins and phenolic chemicals from vegetable tissues, which delivers a high recovery rate with lower treatment duration, decreased energy consumption, and no organic solvents (green extraction). More recently, the authors [13] have also shown that OH has the potential to be used as an efficient and environmentally friendly technology toward sustainable food processes; it has been shown that OH can be used as a pre-treatment for enhanced aqueous extraction of anthocyanins from grape skins, particularly when high-temperature short-time (HTST) treatments are applied. There still is scarce information regarding the effects of combining OH pre-treatments with food-grade solvents to enhance the aqueous extraction of phenolic compounds, namely anthocyanins, comparing with conventional methods based on solvent extraction. Accordingly, this study aimed to evaluate the effectiveness of OH pre-treatments in the aqueous extraction of anthocyanins from red grape pomace by-products using food-grade extraction solvents and compared it with traditional solvent extraction methodologies.

## 2. Results

### 2.1. Total Phenolic Compounds and Antioxidant Capacity

**Figure 1:**
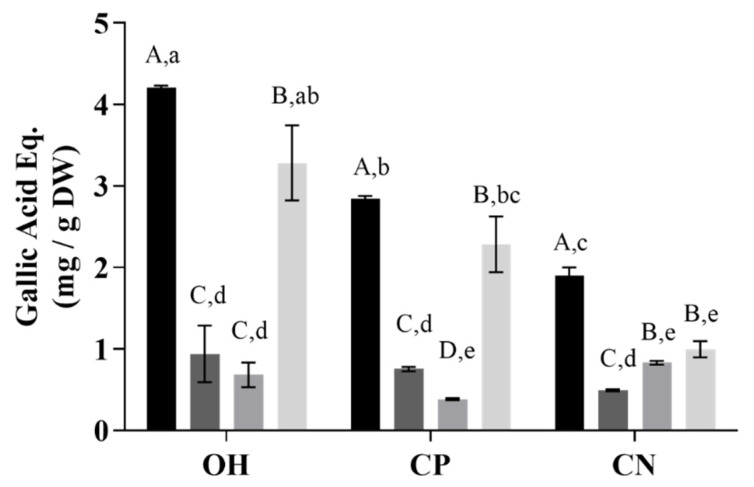
Total phenolic content of extracts performed with methanolic and aqueous solutions from grape wine-making by-products (gallic acid equivalent mg/g DW). OH—ohmic heating; CP—positive control; CN—negative control. Extraction solvents. ▪ MeOH with 1% HCl, ▪ H_2_O, ▪ lactic acid, ▪ citric acid. Different capital letters in the same extraction method indicate a statistically significant difference (*p* < 0.01). The small letters represent statistical differences between solvents and extraction methods (*p* < 0.05).

**Figure 2:**
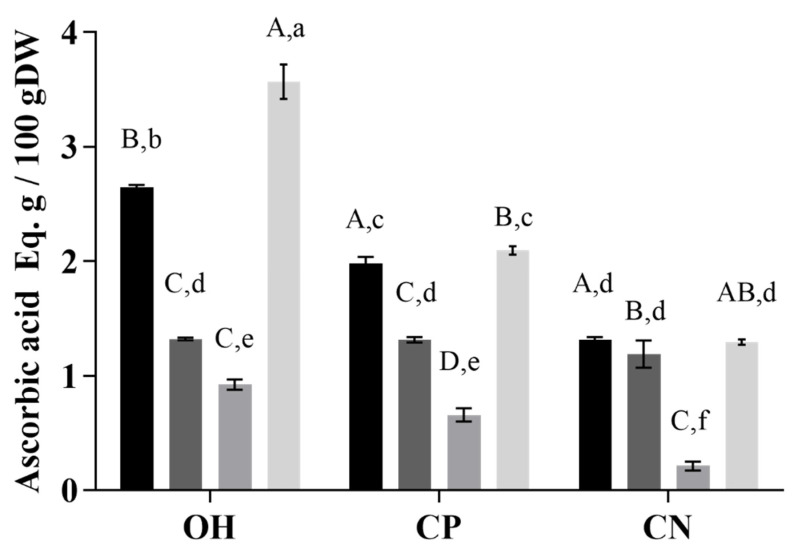
Antioxidant activity by ABTS test of extracts from grape by-products (g ascorbic acid equivalent/100 g DW). OH—ohmic heating; CP—positive control; CN—negative control. Extraction solvents. ▪ MeOH with 1% HCl, ▪ H_2_O, ▪ lactic acid, ▪ citric acid. Different capital letters in the same extraction method indicate a statistically significant difference (*p* < 0.01). The small letters represent statistical differences between solvents and the extraction method (*p* < 0.05).

Concerning total phenolic compounds (TPC) (Figure 1), a better recovery yield was obtained with MeOH acidified in all extraction methods ohmic (OH), negative control (CN), and positive control (CP). Comparing the extraction methods, OH presented higher values than CP for all solvents tested. In addition, higher amounts of TPC were obtained when combined OH with MeOH acidified. Significant differences between extraction methods were only verified for MeOH extraction (*p* < 0.001). In addition, values of 423 ± 0.2 mg/100 g DW gallic acid equivalent and 360 ± 0.8 mg /100 gallic acid equivalent were observed for MeOH and citric acid, respectively, with OH extraction. These results are corroborated by results from antioxidant analysis measured by ABTS, where citric acid showed the highest values of antioxidant capacity (AA) when compared with other solvents (Figure 2).

**Figure 3:**
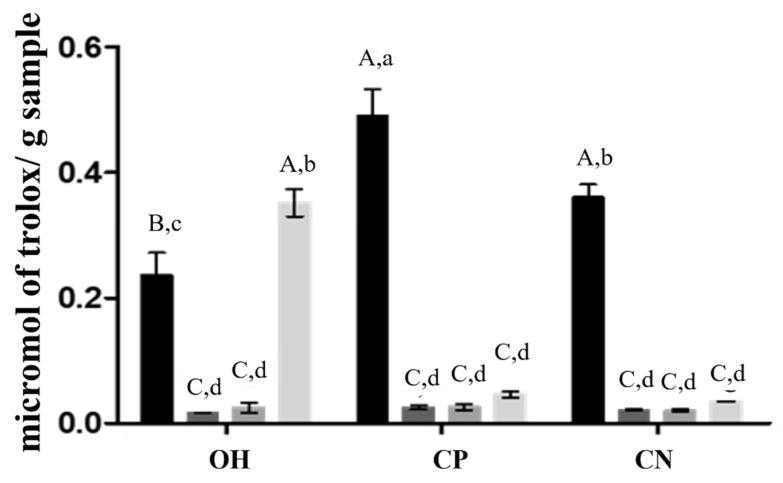
Antioxidant capacity by the ORAC method. Results are expressed in micromol of Trolox equivalent per gram of sample. OH—ohmic heating; CP—positive control; CN—negative control; extraction solvents. ▪ MeOH with 1% HCl, ▪ H_2_O, ▪ lactic acid, ▪ citric acid. Different capital letters in the same extraction method indicate a statistically significant difference (*p* < 0.01). The small letters represent statistical differences between solvents and the extraction method (*p* < 0.05).

AA was also analyzed by the ORAC method. According to the results of AA with the ORAC method presented in Figure 3, this method resulted in different patterns compared with ABTS. Two-way ANOVA showed that there is a significant interaction between solvent and treatment, *p* < 0.0001. Methanol CP extract showed ORAC quantities of 0.489 ± 0.0443 µmol/g Trolox equivalents, which were analogous to those in citric extracts with OH (0.351 ± 0.022 micromol/g Trolox equivalents). 

### 2.2. Total and Individual Anthocyanins Content

**Figure 4:**
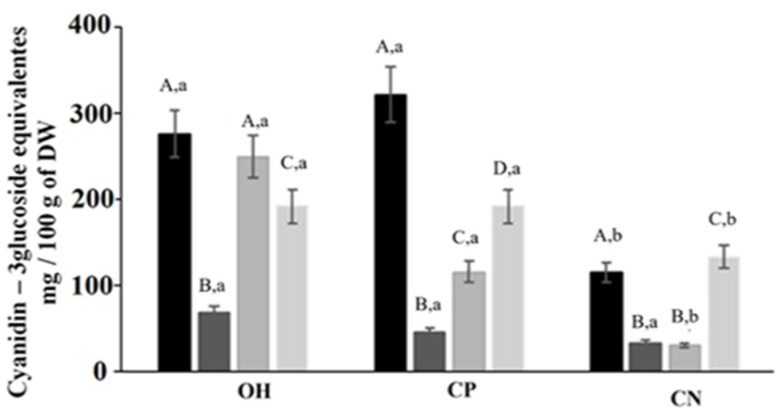
Total anthocyanins for all extraction process of grape by-products. OH—ohmic heating; CP—positive control; CN—negative control. extraction solvents. ▪ MeOH with 1% HCl, ▪ H_2_O, ▪ lactic acid, ▪ citric acid. Different capital letters in the same extraction method indicate a statistically significant difference (*p* < 0.05). Different small letters represent a statistical difference between methods of extraction (*p* < 0.05).

**Figure 5:**
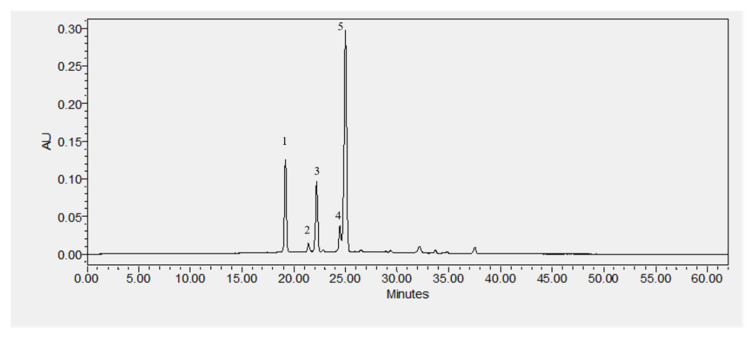
Chromatogram example of anthocyanins profile detected in samples by HPLC-DAD. (1) delphinidin-3-*O*-glucoside; (2) cyanidin-3-*O*-glucoside; (3) petunidin-3-*O*-glucoside; (4) peonidin-3-*O*-glucoside; and (5) malvidin-3-*O*-glucoside.

Results showed an increase of anthocyanin recovery (Figure 4) in OH samples when compared with the CN method for all solvents extraction (*p* < 0.05), while when compared with CP, similar results were obtained. This increase could be explained by non-thermal effects on plant cell permeabilization, which were probably due to electrical disturbances in the membranes of cells or by electroporation impacts [13,15,19]. However, it is essential to highlight that compared with CN, OH treatment resulted in a reduced thermal load. This may have contributed to less degradation of extracted anthocyanins and justify the presented results. Regarding individual compounds, the malvidin-3-*O*-glucoside is the main anthocyanin present for all extracts (Table 1 and Figure 5). 

### 2.3. Antimicrobial Properties

We tested the antimicrobial effects of the extracts using the disk diffusion test and performed a screening of the inhibitory effect on halo formation. The results are presented in Table 2.

Both CP and OH with citric acid extracts exhibited significant higher inhibitory activity against *Escherichia coli* (*E. coli*), *Salmonella enteritidis* (*S. enteritidis*), a methicillin-resistant *Staphylococcus aureus* (MRSA), methicillin-sensitive *Staph. aureus* (MSSA), *Bacillus cereus* (*B. cereus*), *Pseudomonas aeruginosa* (*P. aeruginosa*), and *Yersinia enterocolitica* (*Y. enterocolitica*). It is noteworthy that there are varietal differences involving the phenolic compounds content of grape by-products and, therefore, of their antioxidant and antimicrobial attributes.

Other authors also described that grape extracts at 2% have antibacterial action toward *P. aeruginosa*, *Staph. aureus*, and *E. coli* [20,21,22].

### 2.4. Cytotoxicity

**Figure 6:**
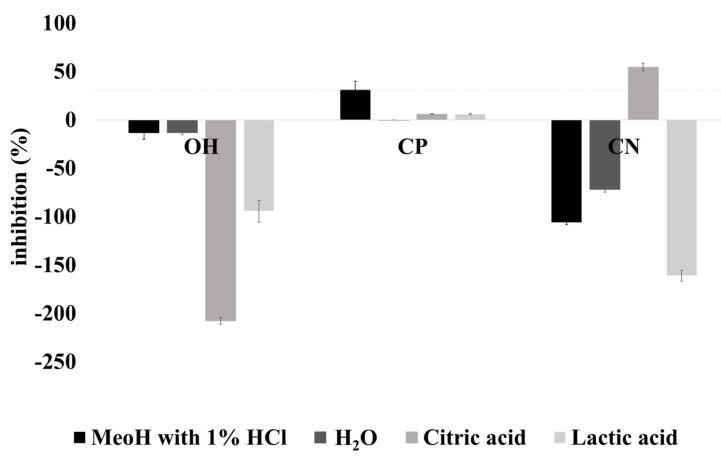
Cytotoxicity analysed by the XTT method. The line represents the non-toxic extracts. OH—ohmic heating; CP—positive control; CN—negative control. The extraction solvents. ▪ MeOH with 1% HCl, ▪ H_2_O, ▪ lactic acid, ▪ citric acid.

The cell viability test XTT (2,3-bis(2-methoxy-4-nitro-5-sulphophenyl)-5-carboxanilide-2*H*-tetrazolium, monosodium salt), a mucus-secreting line HT29-MTX, was utilized to evaluate the potential cytotoxicity effect of grape extracts (OH, CP, and CN). The results showed that the highest concentration of 1 mg /mL of water extracts tested did not inhibit the cellular metabolism (negative values of metabolism inhibition), thus not showing cytotoxicity for these cells (Figure 6). Only citric acid presented an inhibition above 10% for the CN, which indicates some inhibition of cell viability, but also with no relevance, since values are lower than 30% cell metabolism inhibition. All extracts obtained by OH and some from the CN extracts presented negative values, suggesting that they promote cell growth, mainly the extracts produced by OH with citric acid.

## 3. Discussion

### 3.1. Total Phenolic Compounds and Antioxidant Capacity

The results showed that all treatment and solvents influenced the total polyphenols extraction. Significant differences were found between OH and the CN. A decrease in TPC was observed in CN when compared with OH and the CP method. Although OH and CN treatments use high temperatures in the OH, a maximum of 2 s was required for the temperature to reach 100 °C, while in the CT method, 20 min were necessary to reach the same temperature. 

Both heating processes and solvent used affected the TPC. It can cause leaching, and consequently, the TPC can decrease, or it could affect the rupture of the cell wall and, as a result, the release of cell-bound polyphenols. Several studies reported the effect of heating processes on polyphenols content [13,23,24,25,26]. A study carried out by Pereira et al. [13] showed that it is possible to obtain higher extraction yields of TPC and anthocyanins with aqueous extracts with an OH pre-treatment. The fast heating prevents the increase of compounds degradation. Additionally, the polarity of the solution used for polyphenols extraction affects its cell availability, and it also changes the recovery of the TPC. In moderate settings (23 °C), pre-treatments with limited permeabilization effects and no organic solvent have only encouraged the diffusion of small molecular weight hydrophilic components. This explains the differences in the TPC levels across pre-treated OH and CP (*p* > 0.05) samples. In general, the flavonoids in which anthocyanins are contained are low molecular weight molecules and hence easily extracted due to further OH-induced permeabilization.

A significant impact of the treatment was observed when compared to the impact of the solvent by the application of the two-way ANOVA and Tukey’s tests. The treatment accounts for 14.94% of the total variance, with significance *p* < 0.001, while the solvent account for 66.64% of the total variance. The same was observed for AA, according to the ABTS method (Figure 2). Regarding AA, there were differences between results obtained by ABTS and ORAC methods, which could be explained by the sensitivity of methods and the compounds recovered during the extraction processes [27,28]. The ORAC evaluates the AA and determines the antioxidant status in biological systems, while ABTS measures the reduction of the specific force. In addition, different solvents and extraction methods could recover different bioactive compounds, which could justify the differences in values [29].

This result suggested that for TPC and AA, the solvent has more impact than the treatment used. 

Mostly, according to several studies, the inhibition of polyphenol oxidases and acid hydrolysis could occur; as a result, the TPC increases, and consequently, the AA also increases [18,30,31]. The differences found in TPC and AA when citric acid (C_6_H_8_O_7_) or lactic acid (C_3_H_6_O_3_) were used could be explained by its chemical structure and its pH solution, 2.55 and 2.98, respectively. Studies have shown that the nature of the acid employed, along with its pka, influences the selectivity of the extracting medium toward phenolic compounds [32]. Furthermore, the chemical properties of anthocyanins make them susceptible to reactive oxygen molecules.

### 3.2. Total and Individual Anthocyanin Content

Significant differences in total anthocyanin in OH extracts were observed compared to the CP method for food-grade solvents, indicating the greater anthocyanin recovery capacity of red pigments with OH. Anthocyanins are water-soluble glycosides of polyhydroxy and polymethoxy derivatives of 2-phenyl-benzopyryliurn. Furthermore, the pH solution influences the anthocyanin’s colour due to its ionic structure. They present a flavylium form in an acidic condition, being highly soluble in water, and they are also more stable [10,33]. In addition, some studies have shown a disintegration of cell wall caused by electric fields during OH, which could increase the compound’s availability and, consequently, their extraction capacity [15,17,34]. 

Regarding CN, the time to reach 100 °C is significant. In this work, higher levels of anthocyanin degradation were observed in CT samples (exposed to high temperatures for an extended time). These results showed that higher temperatures could promote the degradation of anthocyanins compounds. According to results reported with the literature, the anthocyanins degradation levels could reach 55% with higher temperature usage [35]. Furthermore, some studies also indicate that the heating process and the electric field could potentiate synergies affecting enzymatic activities, such as the polyphenol oxidase activity, which indirectly degrades the monomeric anthocyanins during enzymatic browning [36,37]. Eventually, the electric effects can activate or inactivate enzymes, since there are studies that report effects on the enzymes [38,39,40,41]. However, further studies would be needed to confirm whether the same is true in this case. In addition, the electric fields can promote a selective extraction or even a change in the structure, similar to what happens with proteins [42,43,44,45]. However, it is not known whether the same is true for anthocyanins, and further studies are needed to understand at a fundamental level the interactions between electric fields and anthocyanins molecules.

Regarding individual anthocyanins content, Pereira et al. [13] used water to recover phenolic compounds, and they obtained higher rate yields using OH as pre-treatment to anthocyanins extraction. They only compared OH extraction with the heating process without comparing CONV methods (organic solvents). The authors extracted anthocyanins with OH, which avoids chemical solvents, by using water and reduced treatment times. The main anthocyanins found in this study were malvidin-3-*O*-glucoside, cyanidin-3-*O*-glucoside, and delphinidin-3-*O*-glucoside, which is in agreement with our results. In addition, they present higher cyanidin-3-*O*-glucoside values than delphinidin-3-*O*-glucoside and petunidin-3-*O*-glucoside, while we present lesser cyanidin values compared with delphinidin. The differences found between anthocyanins content could be explained by the extraction method and solvents used. Rackic et al. [46] showed that an alkaline pH increases the cyanidin contents in the extracts, while the opposite happens for pH values ranging from 2.0 and 4.0.

### 3.3. Antimicrobial Properties

Polyphenolic compounds, including anthocyanins, have antimicrobial activity against a wide variety of microorganisms, particularly in inhibiting the development of food-borne pathogens. Anthocyanins demonstrate antimicrobial action through various mechanisms, e.g., causing cell damage by damaging the cell wall, membrane, and intercellular matrix [10]. The amounts of phenolic compounds showing antibacterial action correspond to those previously reported. Polyphenols from Touriga Nacional and Preto Martinho wine by-products were isolated by Silva et al. [47], and they showed strong antibacterial action against different pathogens. These are similar to the findings obtained for the non-conventional extraction OH with water acidified with citric acid, presenting significant differences compared with CN (*p* < 0.05). The CP method displayed similar results regarding antibacterial activity. These microorganisms are usually linked with food as indicators of pathogenic microorganisms. The extraction process of bioactive compounds affects the antimicrobial activity of the recovered compounds [48,49].

Additional investigations with *Staph. aureus* showed that doses as low as 1.6 g/100 g of total phenolics might have a large inhibitory impact on the development of MRSA and MSSA biofilm, but chlorogenic acid was the primary component in this case rather than anthocyanin [50]. The possibility that the OH method could be a selective extraction method for certain compounds, as mentioned above, may potentiate this antimicrobial effect when compared to CN. According to Table 1, the main anthocyanin present in this extract is the malvidin 3- *O*-glucoside. The antibacterial activity of anthocyanin-containing extracts may be caused by the diverse processes and synergy effects of distinct extract phytochemicals such as anthocyanin, phenolic acids, and their chemical combinations [10].

In addition, CN uses a longer thermal effect (100 °C, 20 min). Therefore, it can degrade some more sensitive compounds, including anthocyanins, which have this antimicrobial effect, as described in the literature [51,52]. Other studies have shown that Gram-negative bacteria but not Gram-positive bacteria are inhibited in anthocyanin-rich extracts. This may be related to the distinct cell wall structures between Gram-negative and Gram-positive bacteria in which the outer membrane of Gram-negatives functions as a preventative barrier for hydrophobic compounds but not on hydrophilic compounds [10]. Côté et al. [53] showed the antibacterial activity of cranberry extract in vancomycin-resistant *Enterococcus faecium*, *P. aeruginosa*, *Staph aureus*, and *E. coli*. The antibacterial action of cranberries extracts is not related to their low pH, but it is likely to be attributable to bioactive elements, such anthocyanin and flavonols, in pH-adjusted cranberry extracts.

The results show the potential use of OH extracts against both Gram-negative and Gram-positive bacteria.

### 3.4. Cytotoxicity

The XTT method is an excellent technique for measuring cell viability. Only methanol extracts presented inhibition of cell viability in the case of the CP samples higher than 30%, demonstrating a cytotoxic effect. The results are following the literature, which reports the cytotoxicity of methanolic extracts [54]. In addition, no evidence of cytotoxicity was found with water grape extracts [55]. 

## 4. Materials and Methods

### 4.1. Chemicals 

The 2, 20-azo-bis-(2-methylpropionamidine)-dihydrochloride (AAPH), fluorescein, 2, 2-azinobis-3-ethylbenzothiazoline-6-sulphonic acid (ABTS diammonium salt), potassium sorbate, sodium carbonate, ethylenediaminetetraacetic acid (EDTA), sodium sulfite, and sodium lauryl sulfate were purchased from Sigma-Aldrich (Sintra, Portugal). Methanol, acetonitrile, and hydrochloric acid were purchased from Fischer Scientific Portugal. Folin–Ciocalteu’s reagent, potassium persulfate, citric acid, and lactic acid were purchased from Merck (Algés, Portugal). Standards of ascorbic acid, Trolox, and gallic acid were purchased from Sigma-Aldrich (Sintra, Portugal), while delphinidin-3-*O*-glucoside, cyanidin3-*O*-glucoside, petunidin-3-*O*-glucoside, peonidin-3-*O*-glucoside, and malvidin-3-*O*-glucoside were purchased from Extrasynthese (Lyon, France).

### 4.2. Samples

The red grape pomace, obtained from a wine manufacture using Vinhão cultivar, was used for the study. Grape pomace includes a mixture of pulp, skins, and seeds, which were separated randomly in aliquots of 50 g and dried in an oven at 50 °C ± 3 °C. After samples were milled in a cuisine robot (Bimby Vorwerk, Wuppertal, Germany, TM5), the powder was sieved manually at 150 µm. The powder was used for the following phenolic extractions. 

### 4.3. Extraction Procedures

One of the most critical factors that affects the bioactive compounds recovery yields is the solvents used in the extraction method. Solvents differ in polarity and comprise methanol, hexane, acetone, chloroform, and diethyl ether [56]. According to the literature, the traditional solvent for anthocyanins recovery is acidified methanolic solution [57,58].

#### 4.3.1. Pre-Treatments

**Figure 7:**
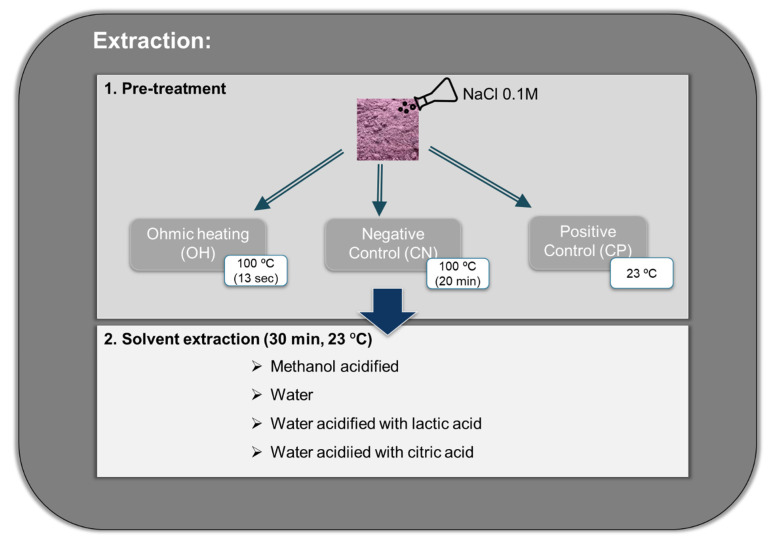
Scheme of experimental analysis.

The grape by-products are non-conductive samples; thus, 2.5 g of grape by-products were placed in 5 mL sodium chloride (NaCl) 0.1 M solution to increase the conductivity to 4.6 mS/cm at room temperature. Three methodologies were performed with each solvent described before—ohmic heating (OH), which reaches 100 °C in 13 s; a control negative (CN) reaches 100 °C in 20 min; and the control positive (CP) used at room temperature (Figure 7)—to understand if the effect came from the ohmic heating (OH) and not from the temperature and solvent during the extraction process. After, all samples were cooled in ice to stop the reactions and proceed with the solvents’ extractions.

##### OH Pre-Treatment

The OH was carried out as a pre-treatment before solvent extraction. A high frequency of 25 kHz and an electric field of ≈30 V/cm was applied. Within these conditions, the temperature reached 100 °C in 13 s, after which the system was turned off; then, samples were placed on ice to stop the residual temperature effect and promote a fast decrease toward room temperature (23 °C). 

##### Control Negative

Control of temperature was also performed to observe the temperature effects. It consisted of placing 2.5 g of grape by-products (placed in the same sodium chloride solution described before) in a bath, and when samples reached 100 °C (≈20 min), they were placed on ice to stop the reaction and also promote a fast decrease toward room temperature (23 °C).

#### 4.3.2. Solvent Extraction

Water, acidified water (lactic and citric acid 1%), and methanol/water solution (80:19:1 *v*/*v*) acidified with hydrochloric acid usually are used as a conventional method (CONV) for anthocyanins extractions, as described by Silva et al. [8]. Both lactic and citric acid are food-grade acids usually used in the food industry. 

Extraction solvents (25.0 mL) were added to pre-treated samples (OH and CN) and directly to 2.5 g of grape by-products pre-treated with NaCl and CP, and the material suspensions were kept under gentle stirring at room temperature (23 °C) for 30 min, allowing the bioactive compound recovery. Water solution and water acidified with two food-grade acids (lactic, citric) were used to decrease the pH (pH 3) to favor extraction and stabilize the color of anthocyanins. 

Then, the extract was centrifuged at 4000× *g*, 4 °C for 10 min, and the supernatant was filtered through a 0.45 mm cellulose acetate filter (Orange Scientific, Braine-l’Alleud, Belgium), and the pellets were stored at −80 °C. This procedure was used for total activities measurement. 

### 4.4. Total Antioxidant Capacity and Phenolic Content

#### 4.4.1. Total Antioxidant Activity 

After the extraction’s procedure, the extracts were evaluated in terms of antioxidant activity to understand the effects of treatments and solvents extraction. 

AA was performed using the ABTS method, according to [14]. The sample was added to a colored solution of 2,2′-azinobis-(3-ethylbenzothiazoline-6-sulfonic acid radical cation) (ABTS^•+^), with an optical density (OD) measured at 734 nm and adjusted to 0.700 ± 0.020 in a spectrophotometric microplate reader (Sunrise Tecan, Grödig, Austria). After 6 min of reaction, the final OD was read, and the results were given in ascorbic acid equivalent. The standard was ascorbic acid (0–500 mg/L), and the regression equation for ascorbic acid and samples was calculated (R^2^ = 0.999). 

The Oxygen Radical Absorbance Capacity (ORAC-FL) was also measured to evaluate the AA, according to Ubeda et al. [45]. Briefly, 20 μL of OH, CN, and CP extracts were mixed with 120 μL of fluorescein (70 nM) in black, untreated 96-well microplates (Nunc, Roskilde, Denmark) and incubated at 40 °C for 10 min. Then, 60 μL of 2,2’-azo-bis-(2-methylpropionamidine)-dihydrochloride (APPH) solution (12 mM, final concentration), Sigma-Aldrich, AAPH solution (60 µL; 12 mM, final concentration) was rapidly added using a multichannel pipet. The microplate was immediately placed in the multidetector plate reader (Synergy H1, Burlington, VT, USA), and the fluorescence was recorded at intervals of 1 min for 140 min. The excitation wavelength was set at 485 nm, and the emission wavelength was set at 528 nm [45]. The microplate was automatically shaken before each reading. The area under the curve (AUC) was calculated for each sample by integrating the relative fluorescence curve. Trolox (10–80 μM) was used as the standard, and regression equations for Trolox and samples were calculated. The ORAC-FL values were calculated by the ratio of sample slope to Trolox slope obtained in the same assay. Final ORAC-FL values were expressed as micromol of Trolox equivalent (TE) per mg of sample.

#### 4.4.2. Total Phenolic Content 

The TPC of extracts was evaluated through the Folin–Ciocalteu spectrophotometric method as described by Ferreira-Santos et al. [59]. A mixture of the sample (5 µL), Folin–Ciocalteu reagent (15 µL), sodium carbonate at 75 g/L (60 μL, Sigma-Aldrich) and distilled water (200 μL) were performed, and the solutions were mixed. Afterwards, samples were heated at 60 °C for 5 min, and the OD was read at 734 nm using a spectrophotometric microplate reader (Sunrise Tecan, Grödig, Austria). The gallic acid standard (0–500 mg/L) was used to measure TPC, and regression equations for gallic acid and samples were calculated (R^2^ 0.997) and expressed as a milligram of gallic acid equivalent per dry weight material (mg GAE/100 g). The analyses were performed in triplicate, and a standard deviation was calculated. 

### 4.5. Total Anthocyanins

Total anthocyanins (TA) were assessed using a spectrophotometric analysis, as Pereira et al. [25] described. The results are expressed in equivalent cyanidine-3-glucoside equivalents (Cy-3-GE) and compared to a range of standards prepared to start from a stock ethanolic solution of cyanidin-3-glucoside. The analysis was performed in triplicate. 

### 4.6. High-Performance Liquid Chromatography (HPLC) Analysis

Polyphenol profiles (quantitative and qualitative) were assessed according to Coelho et al. [14]. The analysis was conducted on HPLC-DAD (Waters Series 600. Mildford MA. USA). A Symmetry^®^ C18 column, 250 × 4.6 mm i.d. 5 μm particle size, and 125 Å pore size with a guard column (waters) was used, and solvents elution consisted of solvent A—acetonitrile (100%) with 0.2% TFA; Solvent B—acetonitrile/water (5:95 *v*/*v*) (Merck pure grade and pure water) with 0.2% TFA (Sigma-Aldrich, Darmstadt, Germany). A linear gradient at a flow rate of 1 mL/min was applied: 0–20 min (100%B); 30–60 min (60% B); and 10 min (100% B). Samples were analyzed in triplicate. Calibration curves were obtained at a detection wavelength of 520 nm. Standards solutions over the concentration range from 0.10 to 100.00 mg/L were prepared for the identification and quantification of the following compounds: delphinidin-3-*O*-glucoside; cyanidin-3-*O*-glucoside; petunidin-3-*O*-glucoside; peonidin-3-*O*-glucoside; and malvidin-3-*O*-glucoside expressed as µg per mL of dry weight (DW) biomass of grape. All calibration curves were linear over the concentration ranges tested with correlation coefficients of 0.999.

### 4.7. Antimicrobial Analysis

#### 4.7.1. Microorganisms

A few pathogenic microorganisms were utilized in this study. Clinical bacterial isolates from urine were kindly given by CHTMAD—Hospital Center of Trás-os-Montes e Alto Douro (through PhD Maria José Alves). The isolated strains comprised *E. coli* (*E. coli* CI resistant to ampicillin, nalidixic acid, norflaxin, and ciproflaxin), a *P. aeruginosa* (*Ps. aeruginosa* CI intermediately resistant to cefotaxime), a methicillin-resistant *Staph. aureus* ((MRSA) resistant to oxacillin, ciprofloxacin, and levofloxacin), and a methicillin-sensitive *Staph aureus* (MSSA) [60]. Additionally, there were three references (R) strains of food isolate from ESB collection: *S*. Enteritidis ATCC 13076, *Y. enterocolitica* NCTC 10406, and *L. monocytogenes*. An inoculum of each bacteria was prepared at a density equivalent to 0.5 on the McFarland scale (~1.5 × 10^8^ CFU mL^−1^). Next, serial decimal dilutions were performed in saline solution, obtaining suspensions with about 1.5 × 10^6^ CFU mL^−1^.

#### 4.7.2. Plate Test 

The different extracts at (1 mg.mL^−1^) were used to evaluate their effects on antimicrobial properties. The assays were performed after 48 and 72 h of sample preparation. To perform these assays, 100 μL of inoculum was transferred to Petri dishes containing the Nutrient Agar medium by the spread plate method, and each plate was incubated at 37 °C for 48 h. As a positive control, nutrient agar plates containing bacterial suspension and saline solution were used as well as plaques containing the bacterial suspension, and the solvents were used to perform the extracts (methanol, water, water acidified with citric and lactic acid). All tests were performed in triplicate according to the method described by Ramos et al. [61]. 

### 4.8. Cytotoxicity

The colorimetric method using the XTT was performed to assess cell viability as a function of redox potential, according to Jiang et al. [62]. In the presence of metabolic activity, the water-soluble XTT is converted to a water-soluble, orange-colored formazan product. Shortly, 100 μL cell suspension aliquots were seeded in a 96 micro-plating well (1 μL to 105 cell/mL) (Nucleon Delta Surface, Thermo Scientific, Roskilde, Denmark). Then, after seeding, the cultivated media was carefully changed and incubated in the dark by the various test solutions. After 24 h, cell viability was tested as follows: a 10 mmol/L of PMS solution was prepared in phosphate-buffered saline (PBS, 0.01 M; pH 7.4), and a 1 mg/mL XTT solution was prepared using the appropriate culture media, previously heated to 37 °C. OH, CN, and CP extracts were used at concentrations of 1.0 mg/mL. Both solutions were sterilized using sterile membrane filters of 0.22 μm (Millipore, Billerica, MA, USA) and combined (2.5 μL PMS per mL XTT solution just before application). In each well, aliquots (25 μL) of mixture were added, and cells were incubated for about 2 h in the dark. The optical density was measured with a microplate reader at 485 nm (FluoSTAR, OPTIMA, BMG Labtech, Ortenberg, Germany). The findings were shown as the percentage of inhibition of cell metabolism. Plain culture media was used as a negative control. All assays were performed in quintuplicate.

### 4.9. Statistical Analysis

All experiments were performed in triplicate, and the results were expressed as the mean ± standard deviation. The SPSS v. 19 (Chicago IL, USA) software was used to evaluate the statistical differences within different treatments determined by ANOVA, using the Shapiro–Wilk for variance homogeneity and Tukey’s test for multiple comparisons. Differences were considered significant at a 5% confidence level (*p* < 0.05).

## 5. Conclusions

The present study assessed the recovery of anthocyanins based on thermal and solvent treatments of grape by-products by OH, CP, and CT extraction methods. The present study assessed for the first time the recovery and characterization of anthocyanins based on thermal treatments of grape by-products by OH combined with acidified food-grade solvents. OH with water acidified with citric acid allowed higher extraction yields of total polyphenols content when compared with other methods. Furthermore, it is possible to obtain extracts with higher AA in the case of OH with water acidified with citric acid than obtained with MeOH. Total anthocyanins recovery was higher with OH and citric acid application. This treatment yielded similar results when compared with the CP method. The main anthocyanins recovery was of malvidin-3-*O*-glucoside, delphinidin-3-*O*-glucoside, and petunidin-3-*O*-glucoside. No cytotoxicity was found for OH extracts obtained with citric acid at 1 mg/mL. On the other hand, for the CP method with MeOH at the same concentrations, there was an inhibition of cell viability of 80%. Additionally, OH with citric acid at 1 mg/mL exhibited antimicrobial properties against pathogens, namely *P. aeruginosa*, *Y. enterocolitica*, *S. enteritidis*, MSSA, MRSA, and *B. cereus*. 

OH combined with food-grade solvent (water and citric acid) allows the recovery of stable anthocyanins, which is in line with the European Union Directive 2009/32/EC. These results demonstrate a relevant opportunity to valorize red grape by-products in a circular economy context. 

## Figures and Tables

**Table 1:** Anthocyanins profile from OH, CONV, and CT methods for each solvent (μg/g DW).

	Extraction Solvent											
	MeOH			Water			Water with Citric Acid			Water with Lactic Acid		
Compounds	OH	CP	CN	OH	CP	CN	OH	CP	CN	OH	CP	CN
delphinidin-3-*O*-glucoside	48.30 ± 0.36A,a	48.82 ± 0.20A,a	20.45 ± 0.09D,b	8.14 ± 0.03F,a	4.90 ± 0.01GH,b	5.08 ± 0.07G,b	36.77 ± 0.81B,a	20.06 ± 0.25D,b	4.71 ± 0.08H,c	29.47 ± 0.49C,a	29.43 ± 0.29C,a	11.87 ± 0.08E,b
cyanidin-3-*O*-glucoside	2.18 ± 0.02C,a	2.94 ± 0.02B,a	0.37 ± 0.07G,b	0.15± 0.01H,c	0.30 ± 0.01G,b	0.49 ± 0.01F,a	1.10 ± 0.08D,a	0.58 ± 0.03E,c	0.70 ± 0.02E,b	0.03 ± 0.003I,b	0.04 ± 0.001I,b	9.79 ± 0.01A,a
petunidine-3-*O*-glucoside	34.24 ± 0.26B,a	38.34 ± 0.16A,b	14.31 ± 0.06D,c	6.66 ± 0.05F,a	4.49 ± 0.06G,b	6.24 ± 0.05F,a	27.46 ± 0.92B,a	0.17 ± 0.01I,b	3.45 ± 0.07H,c	22.10 ± 0.45C,a	22.07 ± 0.45C,a	12.04 ± 0.05E,b
peonidine-3-*O*-glucoside	12.65 ± 0.10B,a	16.86 ± 0.07A,b	5.07 ± 0.02E,c	3.37 ± 0.03F,a	2.32 ± 0.02G,b	1.36 ± 0.02H,c	12.56 ± 0.07B,a	6.43 ± 0.12D,b	0.92 ± 0.06I,c	9.26 ± 0.41C,a	8.92 ± 0.31C,a	2.19 ± 0.20G,b
malvidin-3-*O*-glucoside	128.88 ± 0.94B,b	151.96 ± 0.62A,a	53.41 ± 0.22F,c	37.08 ± 0.06G,a	24.87 ± 0.09H,b	17.34 ± 0.05I,c	125.94 ± 1.25B,a	66.93 ± 1.23E,b	15.57 ± 0.29I,c	101.59 ± 0.39C,a	97.32 ± 0.42C,a	73.02 ± 0.70D,b
Total anthocyanins	224.06 ± 1.25B,b	258.93 ± 2.34A,a	93.62 ± 1.87G,c	55.40 ± 0.99H,a	36.89 ± 1.38I,b	30.51 ± 1.11J,c	203.83 ± 4.21C,a	94.17 ± 1.47G,b	25.35 ± 1.26K,c	162.45 ± 2.14D,a	157.81 ± 1.37E,b	108.90 ± 1.96F,c

Capital letters in rows indicate the significance between treatments (*p* < 0.05). Small letters in rows indicate the differences between solvents of extraction (*p* < 0.05). treatments: OH—ohmic heating; CP—positive control; CN—negative control.

**Table 2:** Antimicrobial activity of extracts (1 mg/mL).

Extraction	Microorganism	OH	CP	CN
MeOH	*Y. enterocolitica*	0	0	0
	*P. aeruginosa*	0	+	0
	*E. coli*	0	0	0
	*S*. *enteritidis*	+	0	0
	*MRSA*	+	0	0
	*MSSA*	0	0	0
	*Listeria monocytogenes*	0	0	0
	*B. cereus*	+	0	0
H_2_O	*Y. enterocolitica*	0	0	0
	*P. aeruginosa*	0	0	0
	*E. coli*	0	0	0
	*S*. *enteritidis*	0	0	0
	*MRSA*	+	0	0
	*MSSA*	0	0	0
	*Listeria monocytogenes*	0	0	0
	*B. cereus*	0	0	0
Lactic acid	*Y. enterocolitica*	0	0	+
	*P. aeruginosa*	0	0	0
	*E. coli*	0	0	+
	*S*. *enteritidis*	0	0	+
	*MRSA*	0	0	+
	*MSSA*	0	0	0
	*Listeria monocytogenes*	0	0	0
	*B. cereus*	0	0	0
Citric acid	*Y. enterocolitica*	+	0	+
	*P. aeruginosa*	+	0	+
	*E. coli*	+	0	0
	*S*. *enteritidis*	+	0	+
	*MRSA*	+	0	+
	*MSSA*	+	0	+
	*Listeria monocytogenes*	0	0	0
	*B. cereus*	++	0	+

Extracts halos for each bacterium (mg/mL) and its inhibitory effect upon disk diffusion test. 0—no halo formation; +—moderate halo formation; ++—strong halo formation.

## Data Availability

The data presented in this study are available on request from the corresponding author.
